# 577. Visual Recognition of GAS Pharyngitis Based on a Photographic Image: A Physician Survey

**DOI:** 10.1093/ofid/ofad500.646

**Published:** 2023-11-27

**Authors:** Rana F Hamdy, Deena Berkowitz, Anqing Zhang, Emily Ansusinha, Jeffrey S Dome, Raj Shekhar

**Affiliations:** Childrens National Hospital, Washington, District of Columbia; Childrens National Health System, Washington, District of Columbia; Childrens National Hospital, Washington, District of Columbia; Children's National Hospital, Washington, District of Columbia; Children’s National Hospital, Washington, District of Columbia; Children's National Hospital, Washington, District of Columbia

## Abstract

**Background:**

Sore throat is one of the most common reasons for an ambulatory pediatric visit in the U.S., and Group A Streptococcus (GAS) pharyngitis is diagnosed in 10-30% of children presenting with pharyngitis. While some prediction rules based on clinical signs and symptoms can reliably identify a low-risk patient, clinical signs or symptoms alone are insufficient in identifying a patient with GAS pharyngitis. The ability to identify GAS from non-GAS pharyngitis from a photographic image has not been previously determined. We conducted a survey to determine clinicians’ ability to identify a GAS vs non-GAS pharyngitis based on a photographic image presented with and without a patient’s clinical data.

**Methods:**

We devised an online electronic survey with photographic images of the posterior pharynx of children presenting with pharyngitis without upper respiratory infection (URI) symptoms who had been evaluated for GAS pharyngitis. The survey included 20 images of the pharynx (10 positive, 10 negative for GAS) with and without clinical data. For each image, participants were asked their prediction for the patient’s GAS test result. We calculated percentage of individual responses correctly identifying GAS vs non-GAS pharyngitis.

**Results:**

102 providers participated in the online survey (23% response rate) and 86 completed all questions in the survey. When counting each response independently, the overall percent of images correctly identified as positive or negative for GAS pharyngitis was 55.9% -- 53% without clinical data, and 59.4% with clinical data (p< 0.001). Among images corresponding to patients who tested positive for GAS pharyngitis, the overall percent correct was 46.6% (38.6% without and 56% with clinical data), and among images corresponding to patients who tested negative for GAS pharyngitis, the percent correct overall was 67.3% (70.5% without and 63.4% with clinical data)(Table 1).

**Figure:**
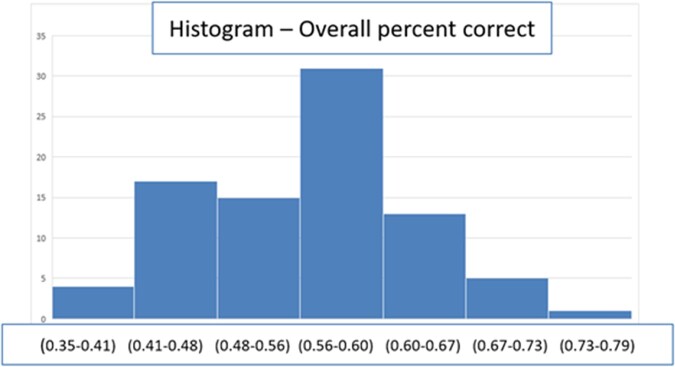

**Table 1:**
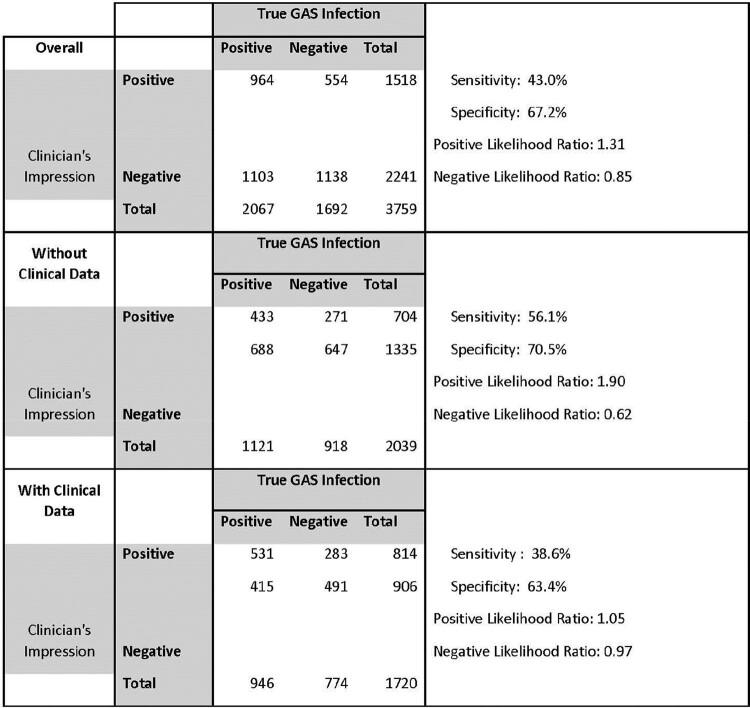
Number of responses from clinician’s impression correct by those with GAS pharyngitis and vs. those without GAS pharyngitis

**Conclusion:**

When presented with a photographic image of the pharynx of a patient presenting with pharyngitis, clinician respondents were able to identify just over half of photographic images as GAS vs non-GAS pharyngitis in children presenting with sore throat and without URI symptoms; this increased modestly when accompanied with clinical data.

**Disclosures:**

**All Authors**: No reported disclosures

